# Augmented 3D super-resolution of fluorescence-free nanoparticles using enhanced dark-field illumination based on wavelength-modulation and a least-cubic algorithm

**DOI:** 10.1038/srep32863

**Published:** 2016-09-13

**Authors:** Peng Zhang, Kyungsoo Kim, Seungah Lee, Suresh Kumar Chakkarapani, Ning Fang, Seong Ho Kang

**Affiliations:** 1 Department of Chemistry, Graduate School, Kyung Hee University, Yongin-si, Gyeonggi-do 17104, Republic of Korea; 2 Department of Applied Mathematics, Kyung Hee University, Yongin-si, Gyeonggi-do 17104, Republic of Korea; 3 Department of Applied Chemistry and Institute of Natural Sciences, Kyung Hee University, Yongin-si, Gyeonggi-do 17104, Republic of Korea; 4 Department of Chemistry, Georgia State University, 308 Petit Science Center, Atlanta, GA 30303, USA

## Abstract

Augmented three-dimensional (3D) subdiffraction-limited resolution of fluorescence-free single-nanoparticles was achieved with wavelength-dependent enhanced dark-field (EDF) illumination and a least-cubic algorithm. Various plasmonic nanoparticles on a glass slide (i.e., gold nanoparticles, GNPs; silver nanoparticles, SNPs; and gold nanorods, GNRs) were imaged and sliced in the z-direction to a thickness of 10 nm. Single-particle images were then compared with simulation data. The 3D coordinates of individual GNP, SNP, and GNR nanoparticles (*x*, *y*, *z*) were resolved by fitting the data with 3D point spread functions using a least-cubic algorithm and collation. Final, 3D super-resolution microscopy (SRM) images were obtained by resolving 3D coordinates and their Cramér-Rao lower bound-based localization precisions in an image space (530 nm × 530 nm × 300 nm) with a specific voxel size (2.5 nm × 2.5 nm × 5 nm). Compared with the commonly used least-square method, the least-cubic method was more useful for finding the center in asymmetric cases (i.e., nanorods) with high precision and accuracy. This novel 3D fluorescence-free SRM technique was successfully applied to resolve the positions of various nanoparticles on glass and gold nanospots (*in vitro*) as well as in a living single cell (*in vivo*) with subdiffraction limited resolution in 3D.

Optical microscopy imaging is very dependable and, as such, is the most widely used analysis method in biomedical and molecular biology research^1^,^2^. However, the spatial resolution of conventional optical microscopy is limited to ~200 nm due to the optical diffraction limitation^3^,^4^,^5^,^6^. To overcome this limitation, several modifications of the point spread function (PSF) and single-molecule localization-based methods were recently developed, including stimulated emission depletion (STED)^7^, ground-state depletion (GSD)^8^, saturated structured illumination microscopy (SSIM)^9^, stochastic optical reconstruction microscopy (STORM)^10^, and photoactivated localization microscopy (PALM)^11^. These techniques were developed to achieve subdiffraction-limited lateral resolution (*x-y*) *in vitro* and *in vivo*.

More recently, with the development of three-dimensional (3D) super-resolution microscopy (SRM), diffraction-limited resolution narrowed to ~10 nm in the lateral dimension and ~20 nm in the axial dimension^12^,^13^. These new techniques provide powerful tools to resolve and analyze the subtle structure of cellular organelles with high-resolution imaging in all three dimensions^14^. The key developments of the 3D SRM techniques were based on modifications and encoding of the PSF in order to resolve the relative central position of the single emitter in the axial direction. Hence, these 3D SRM techniques highly depend on the modification of conventional microscopy using additional optical elements such as cylindrical lenses^15^,^16^,^17^,^18^,^19^,^20^, phase masks^21^,^22^, multi-objective lenses^23^, spatial light modulators^24^,^25^, beam-splitters^26^, adaptive optics devices^27^,^28^, and multi-focus optical elements^29^,^30^,^31^,^32^. The additional optical elements increase the complexity and cost of the instrument and decrease the stability and usability of the 3D SRM imaging system.

Instead of modifying and encoding the PSF in the axial dimension with additional optical elements, optical sectioning methods that employ scanning, such as confocal microscopy^33^, can section the object in the axial direction over time and produce a 3D image of the object. For example, 3D confocal images can be fit to a 3D Gaussian function directly to produce complete 3D SRM images^34^. However, until now, this optical sectioning 3D SRM method has only been successful in confocal based-fluorescence microscopy with fluorescent molecules and quantum dots^35^,^36^. Fluorescent labeling agents add other challenges for the long-term observation of living cells due to photo-bleaching, photo-blinking, and cytotoxicity^37^. Even worse, many cellular targets are difficult to label with fluorophores *in vivo* (such as the mitochondrial-targeted drug delivery carriers) unless complex blended techniques are employed^38^,^39^. In order to overcome these challenges, a fluorescence-free 3D super-localization technique was developed based on differential interference contrast (DIC) microscopy, although DIC microscopy inherently cannot achieve super-resolution^40^. Therefore, a fluorescence-free method for acquiring 3D SRM images over time that maintains the dependability of optical microscopy is sorely needed. Dark-field microscopy, a fluorescence-free based technique, showed the possibility of imaging metal nanoparticles *in vivo* with high signal-to-noise ratio in 3D, especially with specifically designed enhanced dark-field (EDF) devices^41^. However, without the optical control and data fitting process, super-resolution in 3D with EDF is still a challenging issue.

Herein, we describe a least-cubic algorithm and present a novel high precision fluorescence-free 3D SRM method with 10-nm thick slicing in the axial direction. Compared with the commonly used least-square method in SRM, the least-cubic method makes it easier to find the center, especially in asymmetric cases. This method uses direct PSF fitting based on wavelength-dependent EDF illumination detection in 3D and employs a novel least-cubic algorithm without any additional optical elements to enhance accuracy and feasibility.

## Results

### Simulated and experimental 3D SRM images

**Figure 1:**
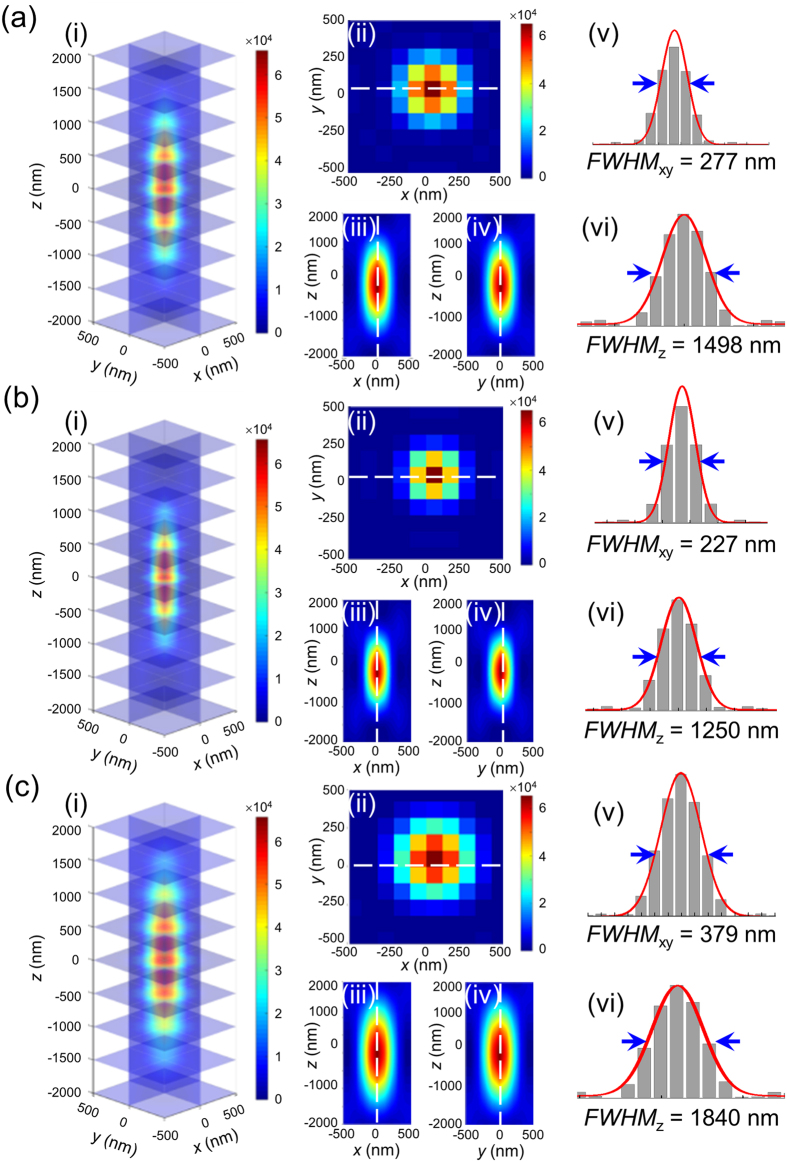
Simulated optical performance of (**a**) GNP, (**b**) SNP, and (**c**) GNR based on the Born-Wolf model. **a**(i), **b**(i), and **c**(i) show the 3D profiles of GNP, SNP, and GNR. **a**(ii), **b**(ii), and **c**(ii) show the *xy* projection at the focal plane of GNP, SNP, and GNR. **a**(iii), **b**(iii), and **c**(iii) show the *xz* projection of GNP, SNP, and GNR; **a**(iv), **b**(iv), and **c**(iv) show the *yz* projection of GNP, SNP, and GNR; **a**(v), **b**(v), and **c**(v) show the *FWHM* in the lateral dimension of GNP, SNP, and GNR. **a**(vi), **b**(vi), and **c**(vi) show the *FWHM* in the axial dimension of GNP, SNP, and GNR.

There are several mathematic models that can be used to simulate the optical profiles of PSF in 3D including the Born-Wolf model, the Gibson-Lanni model, and the Richards-Wolf model^42^,^43^,^44^. Considering the optical profiles of the different models (Supplementary Fig. 1) and the experimental data, the Born-Wolf model was selected to produce 3D simulation EDF microscopy images (see Supplementary Information). For a specimen with a specific refractive index, *n*, and a fixed detection wavelength, *λ*, the PSF shape and size were highly dependent on the numerical aperture (*NA*) of the objective lens (Supplementary Fig. 2). A larger *NA* led to 3D images with a higher spatial resolution. However, due to the basic concepts of dark-field microscopy, the *NA* value of the objective lens must be less than that of the dark-field condenser to prevent the illuminating light from entering the detection system^45^. Therefore, optimization of the *NA* value was necessary for EDF detection. An *NA* of 0.9 was determined to be optimal for EDF detection in our study (Supplementary Fig. 2), and an overly large *NA* value decreased the quality of the images dramatically. In the Born-Wolf model, another factor required to simulate the optical performance in 3D is the wavelength (*λ*) used for imaging. For 103-nm GNP, 80-nm SNP, and 40-nm GNR, the imaging wavelengths corresponded to the localized surface plasmon resonance (LSPR) wavelengths of the nanomaterials (i.e., 575 nm, 473 nm, and 680 nm, respectively)^46^. 3D imaging was then simulated using the optimized *NA* and detection wavelengths (Fig. 1a–c). To quantify the optical performance, the intensity distribution of the PSF was simplified as a 3D Gaussian function:


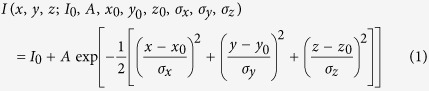


Here, *I*_0_ is a constant term from the background noise, *A* is the amplitude, *x*_0_, *y*_0_, and *z*_0_ are the coordinates of the center, and *σ*_x_, *σ*_y_, and *σ*_z_ are standard deviations of the distribution in the *x*, *y*, and *z* directions. According to the Born-Wolf model, the 3D Gaussian function is an ellipsoid profile with specific widths in the *x*, *y*, and *z* directions (*σ*_x_ = *σ*_y_ ≠ *σ*_z_). Its aspect ratio can be written as:


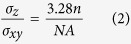


The full width at half maximum (*FWHM*) is calculated as:


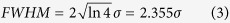


The *FWHM* is the diffraction-limited resolution of dark-field microscopy under ideal conditions. At λ = 575 nm, the *FWHM* in the lateral dimension (*FWHM*_xy_) was 277 nm (Fig. 1a(v)), and in the axial dimension (*FWHM*_z_) it was 1498 nm (Fig. 1a(vi)). At λ = 473 nm, *FWHM*_xy_ was 227 nm (Fig. 1b(v)), and *FWHM*_z_ was 1250 nm (Fig. 1b(vi)). At λ = 680 nm, the *FWHM*_xy_ was 379 nm (Fig. 1c(v)), and the *FWHM*_z_ was 1840 nm (Fig. 1c(vi)).

**Figure 2:**
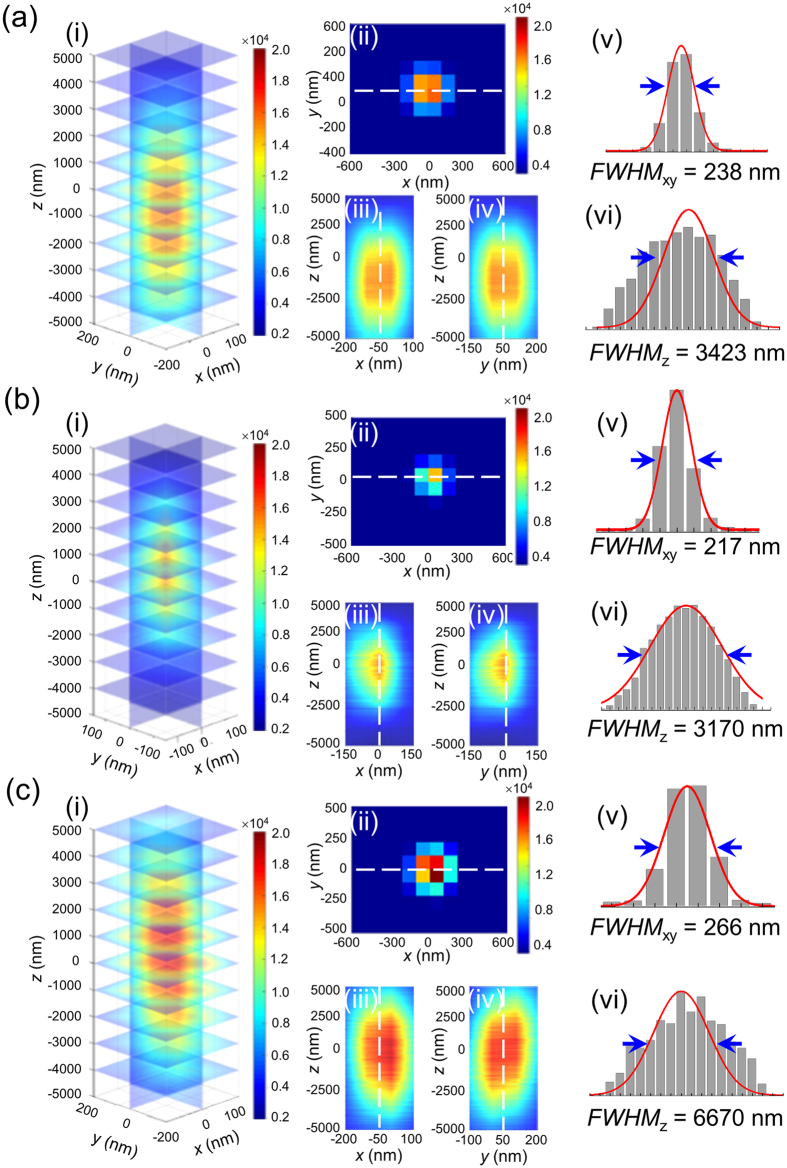
Experimental 3D images of (**a**) GNP, (**b**) SNP, and (**c**) GNR under the EDF system. **a**(i), **b**(i), and **c**(i) show the 3D profiles of GNP, SNP, and GNR. **a**(ii), **b**(ii), and **c**(ii) show the *xy* projection at the focal plane of GNP, SNP, and GNR. **a**(iii), **b**(iii), and **c**(iii) show the *xz* projection of GNP, SNP, and GNR. **a**(iv), **b**(iv), and **c**(iv) show the *yz* projection of GNP, SNP, and GNR. **a**(v), **b**(v), and **c**(v) show the *FWHM* in the lateral dimension of GNP, SNP, and GNR. **a**(vi), **b**(vi), and **c**(vi) show the *FWHM* in the axial dimension of GNP, SNP, and GNR.

The experimental 3D SRM images of GNP, SNP, and GNR on poly-l-lysine (PLL)-coated glass slides at specific plasmonic wavelengths were recorded (Fig. 2). In our previous research, we reported the super-resolution of plasmonic nanoparticles in the lateral dimension^46^, and we used adjacent nanoparticles (lateral distances less than 200 nm) to investigate the resolution in the axial dimension. Figure 2a(v,vi) shows images of GNPs with a 238 nm *FWHM*_xy_ and a 3424 nm *FWHM*_z_. The experimental results were similar to the theoretical predictions in the lateral dimension. However, in the axial direction, the observed intensity distributions were two times larger than the simulated results. Similar discrepancies were observed with SNPs and GNRs (Fig. 2b,c). The extraordinary intensity dispersive component in the axial dimension of the image of every in-focus image plane contains contributions from the neighboring out-of-focus image planes, interference between different imaging slices, and noise from the detection system, all of which are difficult to simulate^33^,^47^,^48^. Although some hardware and mathematical-based methods such as confocal microscopy and deconvolution processes can reduce the contribution of out-of-focus light in each image plane to improve the axial resolution, subdiffraction-limited resolution was not possible^33^,^49^,^50^.

### Augmented 3D super-localization based on Gaussian fitting with a least-cubic algorithm

For 3D super-localization of the nanoparticles, the 3D Gaussian function given by Eq. (1) was fitted based on the given intensity distribution of the PSF with a voxel size of 106 nm × 106 nm × 10 nm (*d*_*x*_ × *d*_*y*_ × *d*_*z*_). The constants (*I*_0_, *A*, *x*_0_, *y*_0_, *z*_0_, *σ*_x_, *σ*_y_, and *σ*_z_) in Eq. (1) were found by applying the least-cubic algorithm to the intensity values (details are provided in the Supplementary Information). Although the least-square algorithm has been the most general and widely used method to find the constants in Eq. (1)^51^, the least-cubic algorithm was proposed in this study due to its ability to handle images of asymmetric particles (i.e., nanorods) with some noise. The developed fitting method was validated with simulated data. Intensity distributions of the PSF were simulated by evaluating the Gaussian function with known constants and adding various amounts of Gaussian noise with a mean of zero and given standard deviations. Then, the constants were inversely estimated using the developed fitting algorithm. The mean differences between the given and estimated particle centers were less than 0.01 *d*_*x*_ (1 nm), 0.01 *d*_*y*_ (1 nm), and 0.5 *d*_*z*_ (5 nm) in the *x*, *y*, and *z* directions, respectively, even with extremely high Gaussian noise (20% of maximum intensity value (Supplementary Figs 3 and 4). Moreover, we cut off the lowest 25% of the intensity values when using the experimental images in order to avoid the influence of background noise (Supplementary Fig. 5 and Table 1). Compared with the commonly used least-square method, the least-cubic method was more useful for finding the center with high precision in asymmetric cases (Supplementary Figs 6 and 7). Finally, the centers of GNP, SNP, and GNR were resolved by applying the fitting methods to empirical intensity distributions.

### Relative position collation

Because the centers of the single-nanoparticles in the axial direction were resolved from the slice positions of each scan, the relative center positions of the individual nanoparticles were collated with the fiducial markers^52^. After collation, the final central positions (in nanometers, relative to the origin point) of the GNP, SNP, and GNR were (312.0, 312.0, 3320.6), (320.8, 219.3, 3314.6), and (234.0, 257.5, 3368.7), respectively with same original point (0, 0, 0).

### Reconstruction of 3D super-resolution images

**Figure 3:**
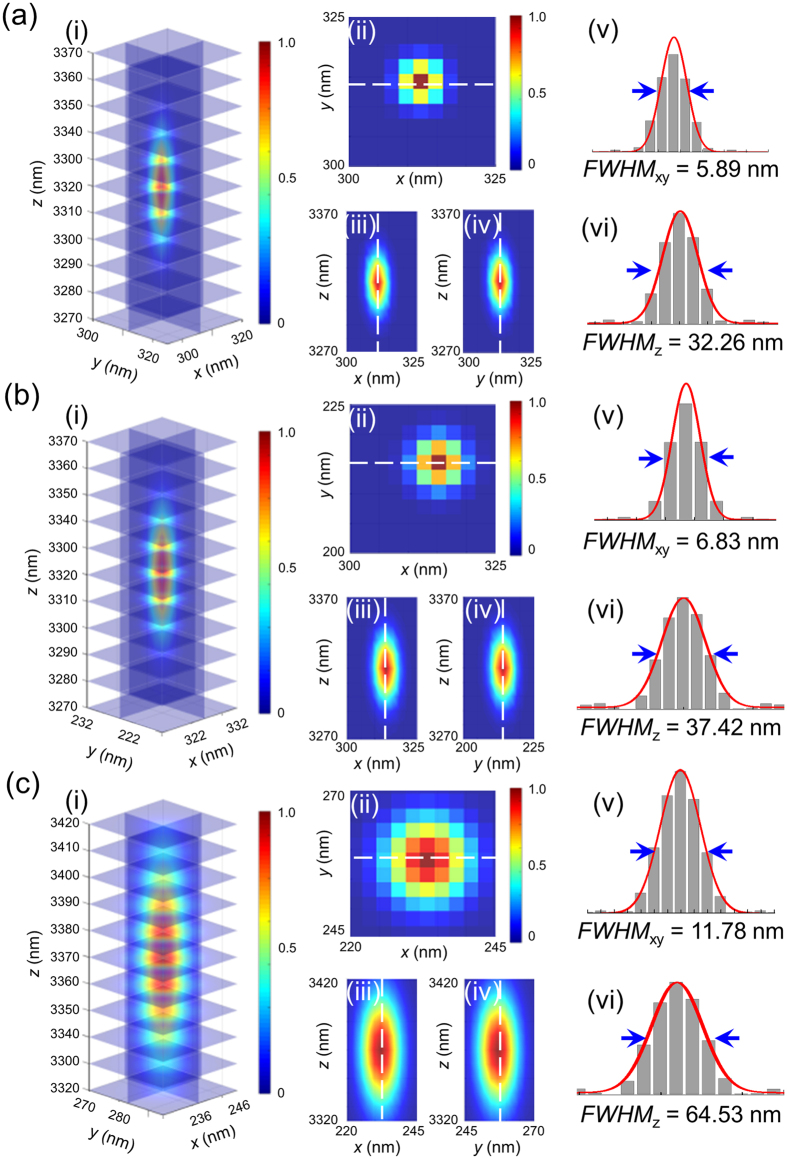
Reconstructed 3D images of (**a**) GNP, (**b**) SNP, and (**c**) GNR under the EDF system. **a**(i), **b**(i), and **c**(i) show the 3D profiles of GNP, SNP, and GNR. **a**(ii), **b**(ii), and **c**(ii) show the *xy* projection at the focal plane of GNP, SNP, and GNR. **a**(iii), **b**(iii), and **c**(iii) show the *xz* projection of GNP, SNP, and GNR. **a**(iv), **b**(iv), and **c**(iv) show the *yz* projection of GNP, SNP, and GNR. **a**(v), **b**(v), and **c**(v) show the *FWHM* in the lateral dimension of GNP, SNP, and GNR. **a**(vi), **b**(vi), and **c**(vi) show the *FWHM* in the axial dimension of GNP, SNP, and GNR. *FWHM* represents the size of the reconstructed images.

**Figure 4:**
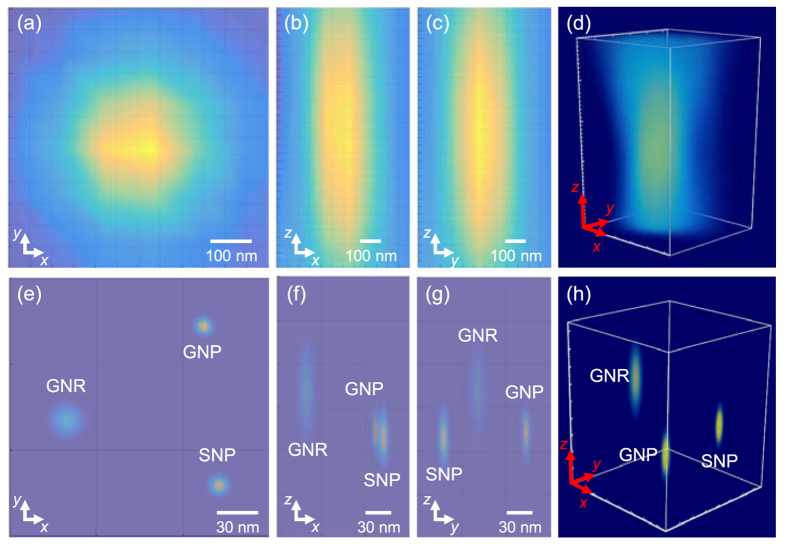
Raw images (**a**–**d**) and reconstructed SRM images (**e–h**) of adjacent GNP, SNP, and GNR on a PLL-coated glass slide. The (**a**,**e**) lateral view (*xy*-view), (**b**,**f**) *xz*-view, (**c**,**g**) *yz*-view, and (**d**,**h**) 3D-view of the nanoparticles.

To reconstruct 3D images of the nanoparticles, we generated Cramér-Rao lower bound (CRLB)-based localization precisions in 3D (*σ*_x_, *σ*_y_ and *σ*_z_). The theoretical precision of a given PSF can be quantified by CRLB with the lowest theoretical localization variance^53^. In our previous research, we showed that lateral localization precisions (*σ*_x,y_) could be measured directly using multiple images^46^. Even though the axial localization precisions (*σ*_z_) could not be measured directly, they could be estimated from Eq. (2) by accounting for the aspect ratio of the ellipsoid Gaussian function. For the GNP at its plasmonic wavelength (575 nm), the optimized lateral localization precision is 2.5 nm^46^, and its axial localization precision *σ*_z_ was estimated to be 13.7 nm. Therefore, the images of GNP with super-resolution were reconstructed as an ellipsoid Gaussian profile with the resolved center (*x*_0_, *y*_0_, *z*_0_) and the measured localizations in lateral and axial dimensions (Fig. 3a). The same approach was applied to SNPs and GNRs, and their CRLB-based localizations were 15.9 nm and 27.4 nm, respectively (Fig. 3b,c). Finally, all three nanoparticles were rendered in one image space to reconstruct the images with super-resolution. The reconstructed 3D SRM images were rendered in an image space (530 nm × 530 nm × 300 nm) with a specific voxel size (2.5 nm × 2.5 nm × 5 nm) (Fig. 4). In the raw images of the three adjacent nanoparticles, the nanoparticles were unresolved in both the lateral (*xy*-direction, Fig. 4a) and axial directions (*xz*- and *yz*-directions, Fig. 4b,c). The raw 3D image of these adjacent nanoparticles did not provide the information required to identify each individual nanoparticle (Fig. 4d). In contrast, after reconstruction, individual GNP, SNP, and GNR particles were resolved in all three dimensions (Fig. 4e–g) and showed subdiffraction resolution. The reconstructed 3D image clearly showed the relative positions of three nanoparticles on the PLL-coated glass slide (Fig. 4h).

### Reconstructed 3D SRM images of nanoparticles on gold nanospots and in living single cells

**Figure 5:**
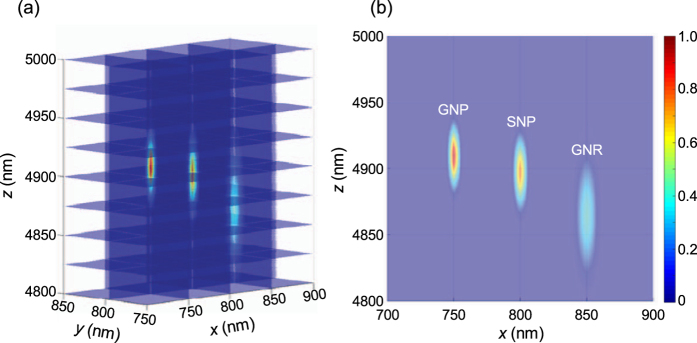
Reconstructed (**a**) 3D SRM images of GNP, SNP, and GNR on a gold nanospot and the (**b**) *xz* projection view.

**Figure 6:**
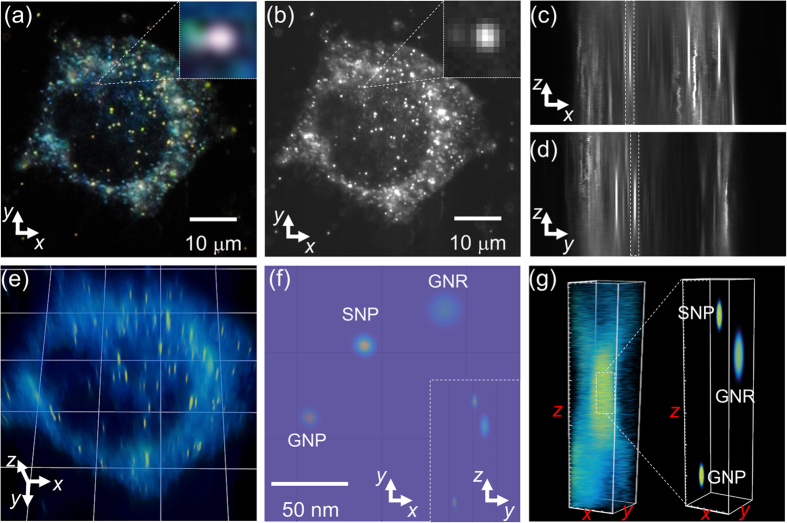
EDF images of GNP, SNP, and GNR in a living single HEK293 cell in (**a**) the *xy*-view with color camera and (**b**) the *xy*-view with CCD camera. The inset figures show the selected NPs used for analysis. (**c**) *xz*-view, (**d**) *yz*-view and (**e**) the 3D view of the same cell. (**f**) Reconstructed SRM images of the selected NPs in lateral (*xy*-view). (**g**) Comparison of the 3D view of raw image (left) and reconstructed 3D SRM images (right) of selected NPs.

3D images of nanoparticles on gold nanospots and in living single cells were reconstructed using our developed augmented 3D super-localization method (Figs 5 and 6). The reconstructed images of NPs on gold nanospots clearly showed differences in the positions of the individual nanoparticles in the axial dimension (Fig. 5) and provided distinct evidence for the conjugation between the NPs and the gold nanospot. Furthermore, 3D SRM images of GNPs, SNPs and GNRs in living human embryonic kidney (HEK) 293 cells were resolved (Fig. 6). Even though the scattering from cell structure was inevitable (Supplementary Fig. 8a), the nanoparticles (Supplementary Fig. 8b) in living cell could be distinguished due to their significant LSPR properties. Since the GNPs, SNPs and GNRs were conjugated to mitochondria *via* anti-mitochondria antibodies, it was impossible to resolve each conjugated nanoparticle even with the highly sensitive enhanced dark-field microscopy (Fig. 6a–d and Supplementary Movies 1 and 2). However, after reconstruction with our 3D SRM technique, three adjacent nanoparticles that were conjugated on the mitochondria were resolved clearly with subdiffraction limit resolution (Fig. 6e–g and Supplementary Movie 3). The relative position of the three NPs in the reconstructed 3D image implied that the GNP and SNP were located at the bottom and top sides of the mitochondria, respectively, while the GNR was located on the side wall of the mitochondria. This successful application of the augmented 3D super-localization method in nanoscience and biology demonstrated that this method was a reliable and universal approach to acquire high precision 3D SRM images of plasmonic nanoparticles *in vitro* and *in vivo*.

## Discussion

In this work, we developed a novel, easy, universal method to achieve 3D SRM imaging of fluorescence-free plasmonic nanoparticles with high precision and without any additional optical elements to modify the PSF. Various nanoparticles including 103-nm GNPs, 80-nm SNPs, and 40-nm GNRs were imaged on glass slides and gold nanospots. EDF illumination was used, and specimens were sliced to a thickness of 10 nm. Due to the inherent limitations of diffraction, differences between individual nanoparticles in the axial dimension were unidentifiable in the raw EDF images. EDF images were selectively observed by applying wavelength modulation that matched with the specific plasmon wavelengths of the nanoparticles. Then, the 3D images of individual nanoparticles were fitted with a 3D Gaussian function based on the least-cubic algorithm to identify the center coordinates (*x*_0_, *y*_0_, *z*_0_). The CRLB values in the lateral and axial dimensions of each nanoparticle were calculated from multiple images. Finally, the 3D images were reconstructed with super-resolution. The method was also applied to distinguish nanoparticle conjugation with gold nanospots and NPs-mitochondria in living single cells. Both cases provided excellent resolution of the nanoparticles in all three dimensions (lateral- and axial-direction). These results demonstrated that this method was a reliable and universal approach to obtain 3D SRM images of plasmonic nanoparticles both *in vitro* and *in vivo*. In addition, unlike the fluorescence labeling method, the scattering of nanoparticles showed highly photo-stability (Supplementary Fig. 10), even with long-term detection period (~10 min). However, some deficiencies still limit the applications of this method. First, in our study, the ~75 nm resolution of adjacent particles was the best condition. The plasmonic coupling between particles played an important role in achieving higher spatial resolution. We believe that novel materials and methods that could avoid the plasmonic coupling effect represent a critical solution. Second, when considered as an axial scanning based method, the temporal resolution of our method was highly dependent on the scanning speed and the number of slices. In general, to obtain high precision 3D images with 10-nm thick slices, ~1000 slices were needed, which took ~10 min. Hence, at present, this method is only suitable for fixed nanoparticles due to the low temporal resolution. In the future, modifications that can improve the temporal resolution should be considered for real-time detection and the study of molecular dynamics. In addition, in the present work, the scattering spectral of nanoparticles only can be estimated from their extinction spectral. The real-time and in suit scattering spectral resolved techniques such as hyperspectral imaging should be a good opinion to improve the optical performance of present work. Furthermore, due to the plasmonic coupling effect of these nanoparticles, it would be challengeable to achieve ultra-high resolution (~10 nm), which is comparable to recently reported fluorescence super-resolution microscopy. We expected the resolution could be improved with the smaller size nanoparticles (i.e., less than 10 nm) in the future.

## Methods

### Chemicals and reagents

The 103-nm GNP, citrate-capped 40-nm GNR (40 nm × 97 nm), and 80-nm SNP colloidal solutions were purchased from Nanopartz^TM^ (Salt Lake City, UT, USA) and BBI Life Sciences (Cardiff, UK). Poly-l-lysine (PLL, 0.01% in purified water), 11-mercaptoundecanoic acid (MUA, 95%), 6-mercapto-1-hexanol (MCH, 97%), 1-ethyl-3-(3-dimethylamino-propyl)carbodiimide hydrochloride (EDC), dimethyl sulfoxide (DMSO, 99.5%), 2-(morpholino)ethanesulfonic acid (MES), glycine, and phosphate buffered saline (PBS) were obtained from Sigma-Aldrich, Inc. (St. Louis, MO, USA). Sulfo-NHS (*N*-hydroxysulfosuccinimide, NHSS), dithiobis(succinimidyl propionate) (DSP), and Protein A/G were purchased from Pierce (Rockford, IL, USA). Tris(base) was purchased from Mallinckrodt Baker, Inc. (Phillipsburg, NJ, USA). StabilGuard was purchased from SurModics (Eden Prairie, MN, USA). Monoclonal antibody (X306) was purchased from HyTest (Turku, Finland). Prior to use, 1× PBS (pH 7.4, 138 mM NaCl, 2.7 mM KCl) and 50 mM MES buffer were filtered through a 0.2-μm membrane filter and photo-bleached overnight using a UV-B lamp (G15TE, 280–315 nm, Philips, The Netherlands).

### Fabrication of 100-nm nanospots

The gold nanospot substrate was fabricated at the National Nanofab Center (Daejeon, South Korea). Briefly, a glass wafer was cleaned using piranha solution (1:1 = H_2_SO_4_:30% H_2_O_2_, v/v). The glass wafer was coated with an electron-sensitive photoresist, such as a 150 nm-thick poly(methyl methacrylate) (PMMA) layer. An electron-beam (Elionix E-beam system, 100 KeV/100 pA) was then used to burn off the polymer in the desired pattern. Au/Cr (20/5 nm thickness) was deposited by thermal evaporation, and PMMA was removed using dichloromethane to form nanospots with a pitch of 10 μm and diameters of 100 nm^54^.

### Electron microscopic images

The size and morphology of nanoparticles (Supplementary Fig. 11) were characterized using an environmental scanning electron microscope (ESEM, Quanta FEG 650, FEI Company, Hillsboro, OR, USA) with an accelerating voltage of 30 kV and a transmission electron microscope (TEM) (2100F, JEOL Ltd, Tokyo, Japan). The samples for ESEM imaging were prepared by dropping a small portion of the sample suspended in distilled water onto a silicon wafer and then drying the wafer in a desiccator. A 5 μL suspension of the nanoparticle mixture (GNPs, GNRs, and SNPs) was dropped onto a Cu-grid (carbon coated, 200-mesh, Ted Pella, Inc., Redding, CA, USA), completely dried, and imaged by TEM.

### Optical properties of nanoparticles and gold nanospots

The extinction spectra of aqueous dispersions of GNPs, GNRs, and SNPs were measured using a UV-Vis spectrometer (MultiSpec-1501, Shimadzu, Tokyo, Japan). The extinction peaks of GNPs, GNRs, and SNPs (Supplementary Fig. 12) were 577 nm, 663 nm, and 477 nm, respectively. The scattering spectra of 100-nm gold nanospots were described previously^54^.

### Immobilizing single nanoparticles on PLL-coated glass

**Figure 7:**
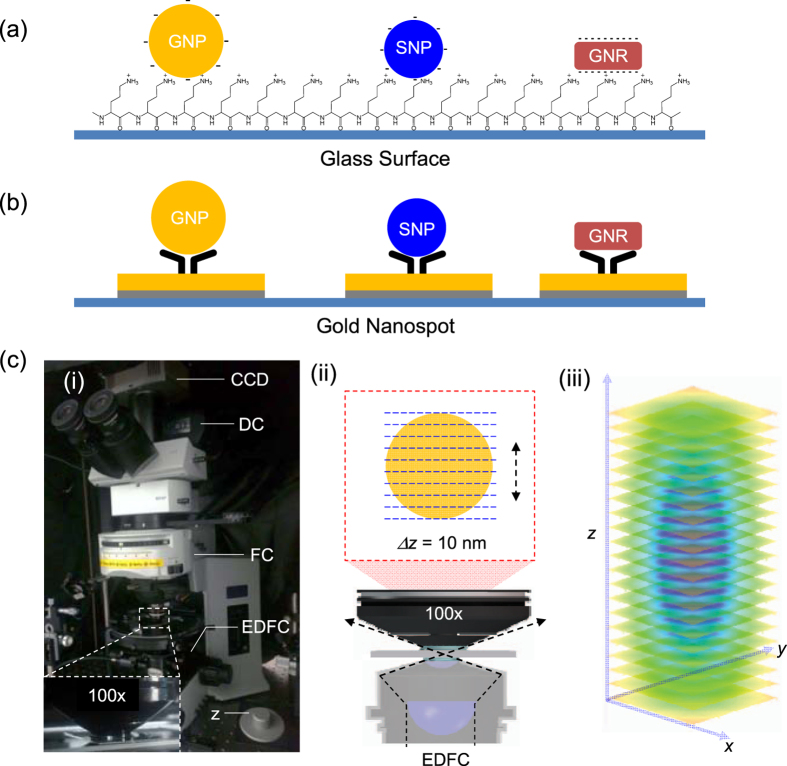
Schematic diagrams of 103-nm GNP, 80-nm SNP, and 40-nm GNR on (**a**) a PLL-coated glass slide, and (**b**) gold nanospots. (**c**) (i) Physical set-up of the EDF illumination based 3D SRM system, (ii) schematic diagram of *z*-slicing, and (iii) the 3D PSF profile. CCD: charge-coupled device; DC: digital camera; FC: filter cube; EDFC: enhanced dark-field condenser; z: *z*-stage controller.

A solution of nanoparticles (3.5 μL, ~10^7 ^particles/mL) was dropped on the PLL-coated glass slide. The negatively charged nanoparticles were immobilized on the positively charged glass surface via electrostatic force (Fig. 7a).

### Conjugation of single nanoparticles on gold nanospots

The single nanoparticles were conjugated on gold nanospots using the following steps. First, the nanoparticles were modified to form NP-MUA-MCH with a carboxylic acid-terminated alkanethiol monolayer on the surface as previously reported^54^. Then, the modified nanoparticles were activated with EDC and Sulfo-NHS to form NP-MUA-MCH-NHSS, as previously reported^54^. Next, the gold nanospots were immersed in 4 mg/mL DSP in DMSO for 30 min and were then rinsed with distilled water. Protein A/G was added at a concentration of 0.1 mg/mL to facilitate the binding of the antibody Fc regions; the solution was incubated for 1 h. Unreacted succinimide groups were blocked by adding 10 mM Tris (pH 7.5) and 1 M glycine and incubating for 30 min. The nanospots were incubated with StabilGuard for 30 min for stabilization then rinsed briefly with a few drops of distilled water. Then, they were incubated with 20 μL of 2 μg/mL antibody (X306) in PBS (pH 7.4) for 1 h. After washing, 20 μL NHS-modified nanoparticles were added to conjugate the nanoparticles on gold nanospots (Fig. 7b and Supplementary Fig. 13), and the reaction was allowed to proceed for 2 h. The scattering spectra of the nanoparticles showed some red-shift due to the plasmonic coupling of nanoparticles and the gold nanospot, especially for the SNP (~40 nm shift)^55^.

### Conjugation of nanoparticles with antibody

The GNP, SNP and GNR were conjugated with anti-mitochondria antibodies (MAB 1273, Millipore, Schwalbach, Germany) as follows. Exactly 10 mM MUA and 30 mM MCH dissolved in ethanol were added to a nanoparticle solution suspended in water, followed by sonication for 10 min and incubation for 2 h 30 min to form a self-assembled monolayer (SAM) of MUA-MCH on the NPs surface. The monolayer was then washed with ultra-pure water by centrifugation (12 000 rpm, 90 min, 4 °C). The NPs-MUA-MCH was re-suspended in 50 mM MES and 0.1 M NaCl (pH 6.0). These procedures allowed for the formation of a carboxylic acid-terminated alkanethiol monolayer on the surface of the NPs. Conjugation of NPs with antibody was carried out using EDC and Sulfo-NHS. EDC (40 μg, 2 mg/mL in 50 mM MES pH 6.0) and Sulfo-NHS (196 μg, 2 mg/mL in 1 × PBS, pH 7.4) were added to the NPs-MUA-MCH solution. After stirring at room temperature for 40 min, NPs-MUA-MCH-NHSS was washed using centrifugation (17 000 rpm, 30 min, 4 °C) and then re-dissolved in PBS. The anti-mitochondria antibody solution was diluted 400 times in PBS (pH 7.4), added to the activated GNP, SNP, and GNR, and allowed to react using a rotary shaker for 4 h at room temperature. The mixture was then stored for 12 h at 4 °C. Tris (10 mM, pH 7.5) and 1 M glycine were added and the solution was incubated for 15 min to quench the excess hydroxylamine. The final nanoparticle-antibody suspension was centrifuged (17 000 rpm, 90 min, 4 °C) to remove any unbound antibody and re-suspended in PBS buffer and stored at 4 °C.

### Incubation of nanoparticles with living cells

Cultures of HEK293 cells were plated as previously described^56^ and were grown in cell culture medium. Cells were maintained in plastic tissue culture dishes (BD Biosciences, Bedford, MA, USA) at 37 °C with a humidified atmosphere containing 5% CO_2_. For single-cell imaging, cells were placed on a 22 mm × 22 mm cover-glass (No. 1, Deckglaser, Freiburg, Germany) and were incubated for 24 h. Adherent cells were rinsed twice with Dulbecco’s phosphate buffered saline (DPBS), and then were immediately exposed to the medium containing antibody conjugated GNPs, SNPs, and GNRs followed by incubation for 24 h. Cover glasses with adherent cells were washed 3 times with DPBS to remove excess NPs and were then placed under the objective lens to obtain images.

### Lab-built 3D wavelength-dependent SRM system with *z*-motor

A lab-built EDF illumination microscopic system (Fig. 7c(i)) was constructed using an Olympus BX-51 upright microscope (BX-51, Olympus, Tokyo, Japan) equipped with a CytoViva EDF illumination device (CytoViva Inc., Auburn, AL, USA) and a 100× objective lens (UPLANFLN, adjustable *NA* from 0.6 to 1.3, Olympus). *Z*-axis sectioning with 10-nm thickness was achieved using a *z*-motor (LEP MAC 5000, LUDL Electronic Products Ltd., Hawthorne, NY, USA) to obtain the 3D information of the specimen. An electron multiple charge-coupled device (EMCCD) camera (QuantEM, 512 SC, Photometrics, Tucson, AZ, USA) and a colored Nikon D3S digital camera (Tokyo, Japan) were installed on the top of the microscope to detect single-particle images simultaneously. Band-pass filters with various wavelengths (473 ± 10 nm, 575 ± 15 nm, and 680 ± 10 nm) purchased from Semrock (Rochester, NY, USA) were used to select the detected scattering wavelength of the specimen. MetaMorph (Universal Imaging, Sunnyvale, CA, USA), the ThunderSTORM plug-in of ImageJ (NIH)^52^, and a lab-built least-cubic 3D data fitting program based in Matlab (Mathworks, Torrance, CA, USA) were used for image acquisition and data processing.

### 3D EDF imaging of specimen

The specimen (nanoparticles on the PLL-coated glass slide, nanoparticle-conjugated gold nanospot, and nanoparticle-conjugated antibody in living cells) was fixed on the *z*-motor driving stage, and moved along the axial direction in 10-nm increments to acquire 3D information of the single nanoparticles at their specific LSPR wavelengths (Fig. 7c(ii,iii)). Because the Gaussian function in the axial direction is symmetric, the relative positions of individual particles were not unique. Thus, to avoid the ambiguity of the relative positions, the scanning direction of every cycle was the same (from top to bottom), and the relative position information could be resolved from the slice order.

## Additional Information

**How to cite this article**: Zhang, P. *et al*. Augmented 3D super-resolution of fluorescence-free nanoparticles using enhanced dark-field illumination based on wavelength-modulation and a least-cubic algorithm. *Sci. Rep*. **6**, 32863; doi: 10.1038/srep32863 (2016).

## Supplementary Material

Supplementary Information

Supplementary Movie S1

Supplementary Movie S2

Supplementary Movie S3
