# Supplementary material for: Augmented 3D super-resolution of fluorescence-free nanoparticles using enhanced dark-field illumination based on wavelength-modulation and a least-cubic algorithm
Source: Sci Rep. 2016 Sep 13;6:32863. doi: 10.1038/srep32863 (PMC5020655; doi:10.1038/srep32863)
Supplement: Supplementary Information [file srep32863-s1.pdf]

Supplementary Information

Augmented 3D super-resolution of fluorescence-free nanoparticles using enhanced dark-field illumination based on wavelength-modulation and a least-cubic algorithm

Peng Zhang[1], Kyungsoo Kim[2], Seungah Lee[3], Suresh Kumar Chakkarapani[1], Ning Fang[4], and Seong Ho Kang[1,3,*]

[1]Department of Chemistry, Graduate School, Kyung Hee University, Yongin-si, Gyeonggi-do 17104, Republic of Korea

[2]Department of Applied Mathematics, Kyung Hee University, Yongin-si, Gyeonggi-do 17104, Republic of Korea

[3]Department of Applied Chemistry and Institute of Natural Sciences, Kyung Hee University, Yongin-si, Gyeonggi-do 17104, Republic of Korea

[4]Department of Chemistry, Georgia State University, 308 Petit Science Center, Atlanta, GA 30303, USA

***Correspondence and requests for materials should be addressed to S.H.K. (email: shkang@khu.ac.kr).

The AVI movie “Supplementary Movie 1” shows the EDF images of GNP, SNP, and GNR in a living HEK293 cell with *z*-slicing. The slice interval was 10 nm, and the NPs were conjugated with anti-mitochondria antibody (MAB1273).

The AVI movie “Supplementary Movie 2” shows the 3D rotation view of EDF images of GNP, SNP, and GNR in a living HEK293 cell.

The AVI movie “Supplementary Movie 3” shows a comparison of raw images and reconstructed 3D-SR images of adjacent NPs in a living HEK293 cell.

Basic theory of 3D diffraction optics.

The 3D PSF of wide-field microscopy was demonstrated by the Born-Wolf model[1-3]. This model describes the scalar-based diffraction that occurs in the microscope but neglects the aberrations of the refractive index (n) from the immersion medium and the substrate.

$$PSF(x,y,z) = -\frac{2\pi i a^2 A}{\lambda f^2} e^{i\left(\frac{f}{a}\right)^2 \mu} \int_0^1 e^{-\frac{i\mu\rho^2}{2}} J_0(v\rho)\rho d\rho \quad (1)$$

Here, $\mu = \frac{2\pi}{\lambda}\left(\frac{a}{f}\right)^2 z$, $v = \frac{2\pi}{\lambda}\left(\frac{a}{f}\right) r$, $r = \sqrt{x^2 + y^2}$, $\frac{a}{f} = \frac{NA}{n}$, and $\rho = \frac{r}{a}$; $J_0(v\rho)$ is the Bessel function of zero order; r, a, and f are the co-ordinates, the radius of the exit pupil, and the focal distance of the objective, respectively. λ is the wavelength of light, n is the refractive index of the object medium, NA is the numerical aperture of the objective lens, and A is the amplitude factor. The intensity distribution of the diffraction pattern in 3D is described as

$$I(x,y,z) = \left|PSF(x,y,z)\right|^{2N+2} \quad (2)$$

For dark-field microscopy, N is 0. Therefore,

$$I(x,y,z) = \left|PSF(x,y,z)\right|^{2} \quad (3)$$

At the geometrical focus, $\mu = v = 0$ and the intensity is

$$I_0 = \frac{1}{4}\left(\frac{ka^2 A}{f^2}\right) \quad (4)$$

For the point in the focal plane, $\mu = 0$ and the intensity distribution is

$$I_{xy} = I_0 \left(2\frac{J_1(v)}{v}\right)^2 \quad (5)$$

Optimization based on the least-cubic algorithm.

Let the following 3D Gaussian function be the intensity distribution of PSF.

$$I(x,y,z;I_0,A,x_0,y_0,z_0,\sigma_x,\sigma_y,\sigma_z)$$
$$= I_0 + A\exp\left[-\frac{1}{2}\left[\left(\frac{x-x_0}{\sigma_x}\right)^2 + \left(\frac{y-y_0}{\sigma_y}\right)^2 + \left(\frac{z-z_0}{\sigma_z}\right)^2\right]\right] \quad (6)$$

In addition, let $I_{\exp}(x,y,z)$ be the intensity value at the location (x,y,z) obtained from the experiment. Assume that the intensity values are observed in a rectangular parallelepiped volume $V = \{(x,y,z)|\ a \le x \le b,\ c \le y \le d,\ e \le z \le f\}$.

We found the constants (I_0, A, x_0, y_0, z_0, σ_x, σ_y, and σ_z) to minimize the objective function $F(I_0,A,x_0,y_0,z_0,\sigma_x,\sigma_y,\sigma_z)$ when p was 2 or 3:

$$F(I_0,A,x_0,y_0,z_0,\sigma_x,\sigma_y,\sigma_z)$$
$$= \sum_{(x,y,z)\in V} \left|I(x,y,z;I_0,A,x_0,y_0,z_0,\sigma_x,\sigma_y,\sigma_z) - I_{\exp}(x,y,z)\right|^p \quad (7)$$

The case of $p = 2$ implies the conventional least-square algorithm, and the case of $p = 3$, called the least-cubic algorithm, is useful for finding the constants when considering noises and asymmetric cases. In this study, the least-cubic algorithm was implemented after its validity was guaranteed by testing the Gaussian function of known constants with Gaussian noise up to 20% of the maximum intensity value (51 of 255) together with the least-square algorithm (Supplementary Figs. 3 and 4). The mean and maximum differences between the given and estimated particle centers were less than 0.01 d_x (1 nm) and 0.03 d_x (3 nm), 0.01 d_y (1 nm) and 0.04 d_y (4 nm), and 0.5 d_z (5 nm) and 1.0 d_z (10 nm) in the x, y, and z directions, respectively, both in the least-cubic and least-square algorithm (Supplementary Fig. 4). In addition, we cut

off the low-level intensity values to less than an empirically established threshold (w) when using the experimental images in order to avoid the influence of noise (Supplementary Fig. 5):

$$\bar{I}_{\exp}(x,y,z) = \mathrm{Max}\left(\frac{I_{\exp}(x,y,z) - I_{\mathrm{Min}}}{I_{\mathrm{Max}} - I_{\mathrm{Min}}}, w\right) \times \frac{w}{1-w} \qquad (8)$$

Here, $I_{\exp}(x,y,z)$ is the intensity value at (x,y,z) in the obtained image, I_{Max} is the maximum intensity value in the obtained image, I_{Min} is the minimum intensity value in the obtained image, and $\bar{I}_{\exp}(x,y,z)$ is the normalized intensity value at (x,y,z) after cutting off (Supplementary Fig. 5). We then substituted $I_{\exp}(x,y,z)$ by $\bar{I}_{\exp}(x,y,z)$ in Eq. (7). We tested threshold values (w) from 0% to 30% for the least-cubic and least-square algorithms using experimental images of three particles; threshold values higher than 30% might lead to the removal of meaningful intensity values. The threshold value of approximately 20-30% provided reasonable results for both algorithms. Moreover, the least-cubic algorithm more quickly achieved a stable value as the threshold value increased. The centers with 0-5% cut-off were closer to the centers with 25-30% cut-off in the least-cubic method than the least-square method (Supplementary Table 1). In addition, we tested GNP, SNP, and GNR particles to find their centers (Supplementary Fig. 6). Using the GNP and SNP particles that have quite symmetric shapes, the centers obtained using the least-cubic algorithm were very similar to those obtained using the least-square algorithm. However, the center of the GNR was dependent on the algorithm, and the difference might have originated from the asymmetric shape (Supplementary Fig. 6 the TEM image of GNR). We also tested another asymmetric image to find the center of a particle. The

image was obtained by removing intensity values of the upper 200 slices of simulated 3D EDF images of GNP with no Gaussian noise, used in Supplementary Fig. 3. The center was predicted at (0.0 d_x, 0.0 d_y, -20.7 d_z) and (0.0 d_x, 0.0 d_y, -23.6 d_z) by the least-cubic and least-square algorithms, respectively, while the original center was located at (0 d_x, 0 d_y, 0 d_z) (Supplementary Fig. 7). This result indicated that the center found by the least-cubic algorithm was closer to the brightest spot than the center found using the least-square algorithm. Therefore, the assumption that the particle center is located at the brightest spot in an ideal case could imply that the least-cubic algorithm was appropriate to estimate the center of a given particle, even for the nanoparticles at various conditions (i.e., on PLL-coated glass slide, on gold nanospot, and in living single HEK293 cell) (Supplementary Fig. 9).

Supplementary Table 1. Examples of estimated center coordinates by the least-cubic and the least-square algorithms for various cut-off thresholds (*w*) using experimental images of three particles (Unit: pixel).

	Least-cubic algorithm				Least-square algorithm			
	w	*x*	*y*	*z*	*w*	*x*	*y*	*z*
Particle 1	0%	5.1	6.2	173.3	0%	5.3	6.1	202.9
	5%	4.9	6.2	159.1	5%	5.0	6.2	168.3
	10%	4.8	6.2	153.9	10%	4.9	6.2	155.8
	15%	4.8	6.2	152.4	15%	4.9	6.2	153.2
	20%	4.8	6.2	151.7	20%	4.9	6.2	152.4
	25%	4.8	6.3	151.4	25%	4.9	6.3	152.1
	30%	4.8	6.2	151.7	30%	4.9	6.3	151.8
Particle 2	0%	5.2	6.9	187.4	0%	5.4	6.8	242.9
	5%	5.0	6.9	160.2	5%	5.1	7.0	169.1
	10%	4.8	6.9	145.5	10%	4.9	7.0	147.3
	15%	4.8	6.8	140.7	15%	4.8	6.8	140.2
	20%	4.8	6.6	142.3	20%	4.8	6.6	142.3
	25%	4.8	6.5	145.2	25%	4.8	6.5	144.7
	30%	4.8	6.5	147.3	30%	4.8	6.5	146.5
Particle 3	0%	8.3	7.5	553.4	0%	8.2	7.6	500.3
	5%	8.3	7.5	566.5	5%	8.4	7.5	510.1
	10%	8.3	7.5	581.4	10%	8.3	7.5	585.1
	15%	8.3	7.5	596.1	15%	8.3	7.5	598.9
	20%	8.3	7.5	605.7	20%	8.3	7.5	607.0
	25%	8.4	7.5	610.5	25%	8.4	7.5	611.2
	30%	8.4	7.5	612.9	30%	8.4	7.5	612.9

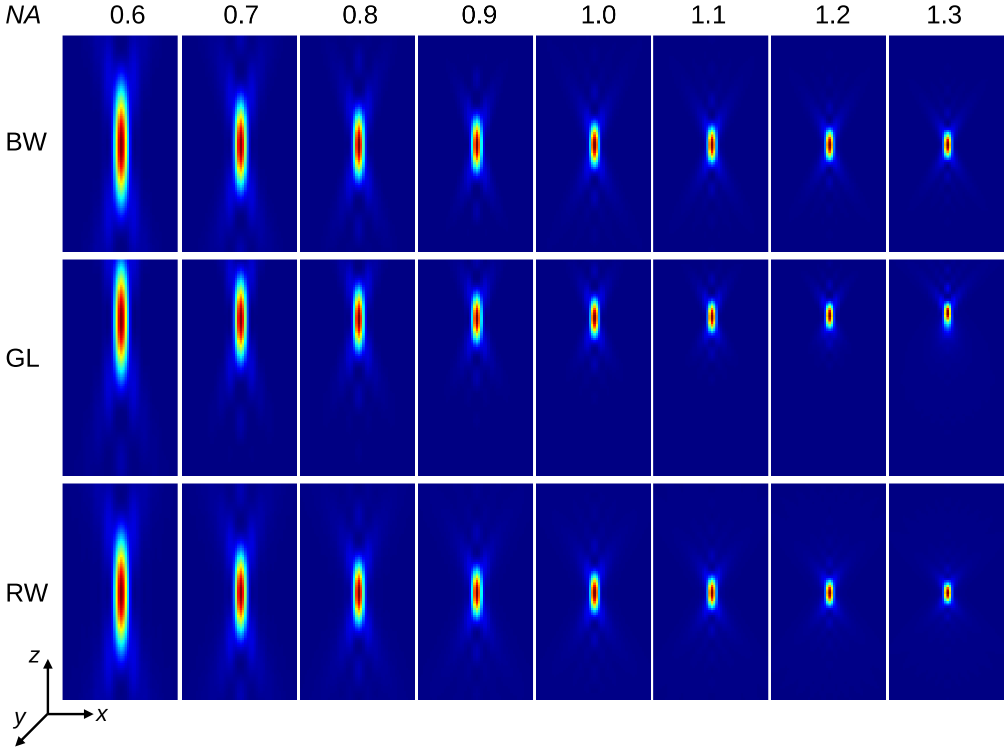

Supplementary Figure 1. 3D image profiles of the Born-Wolf (BW) model, Gibson-Lanni (GL) model, and Richards-Wolf (RW) model with various *NA* values.

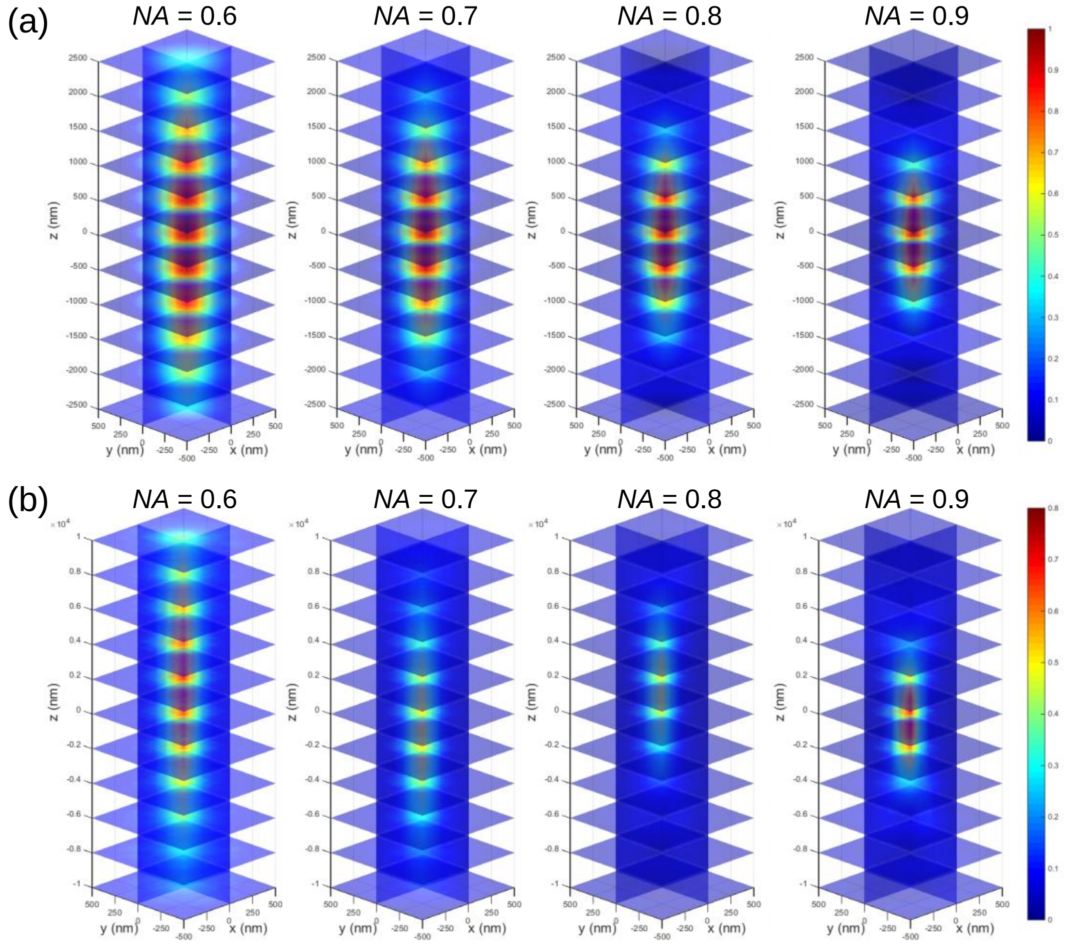

Supplementary Figure 2. (a) Born-Wolf model-based simulation and (b) experimental 3D microscopy images with various *NA* values.

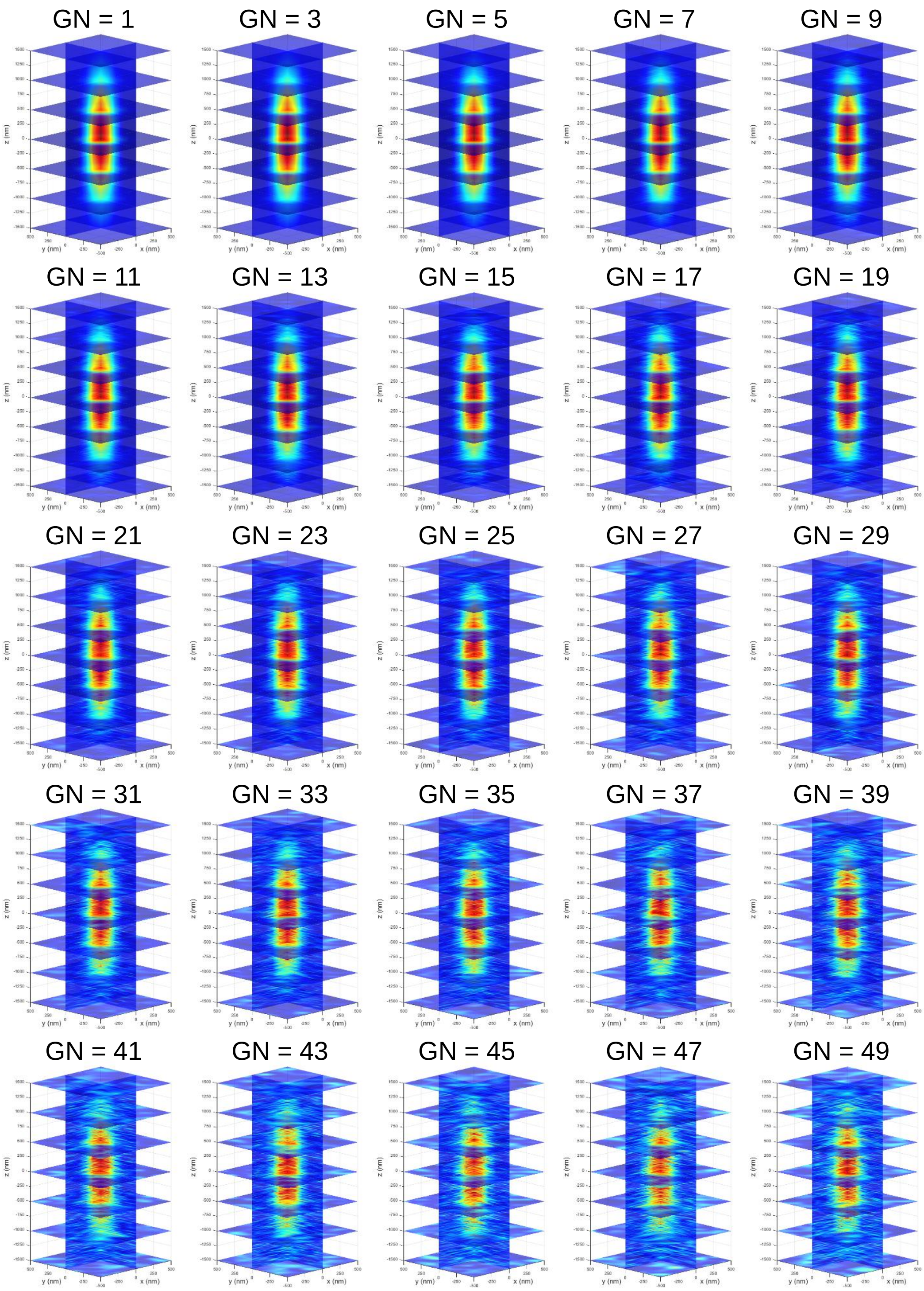

Supplementary Figure 3. Simulated 3D EDF images of GNPs with various Gaussian noise (GN).

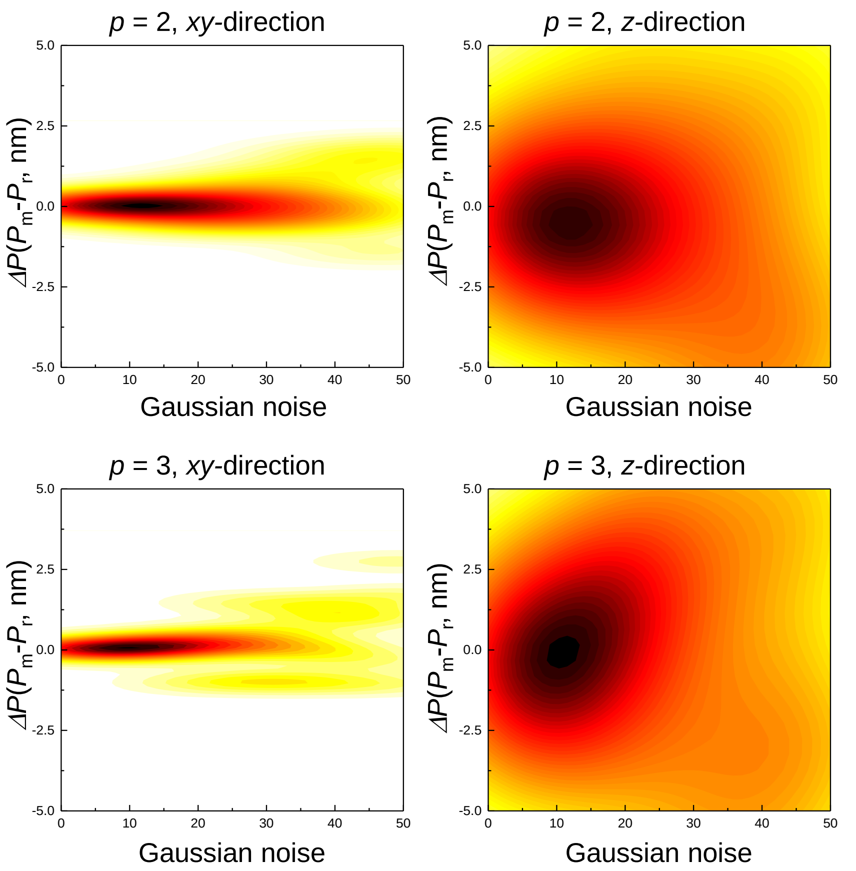

Supplementary Figure 4. Center localization error distributions in the lateral direction (*xy*) and the axial direction (*z*) with various amounts of Gaussian noise by the least-square (p = 2) and least-cubic (p = 3) fitting algorithms.

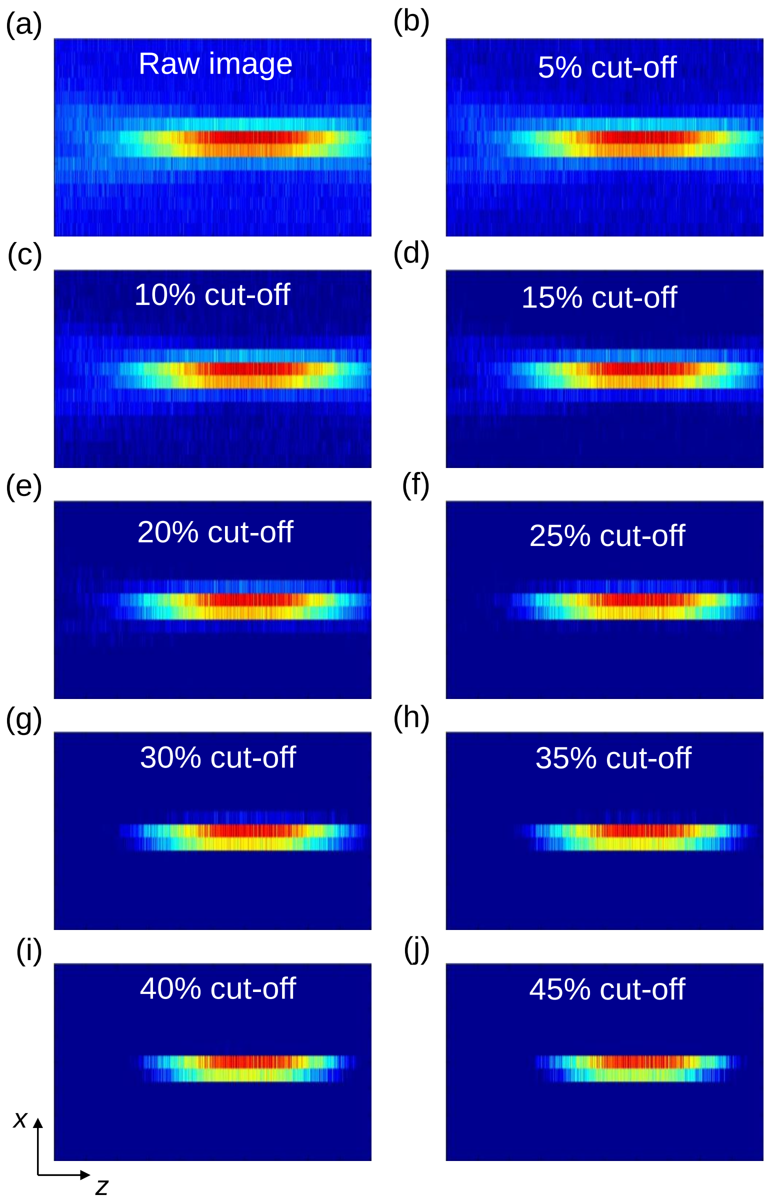

Supplementary Figure 5. An example of a raw image and its lower intensity cut-off images. (a) Raw image and (b-j) images after cut off with various intensity threshold values (w = 5-45%).

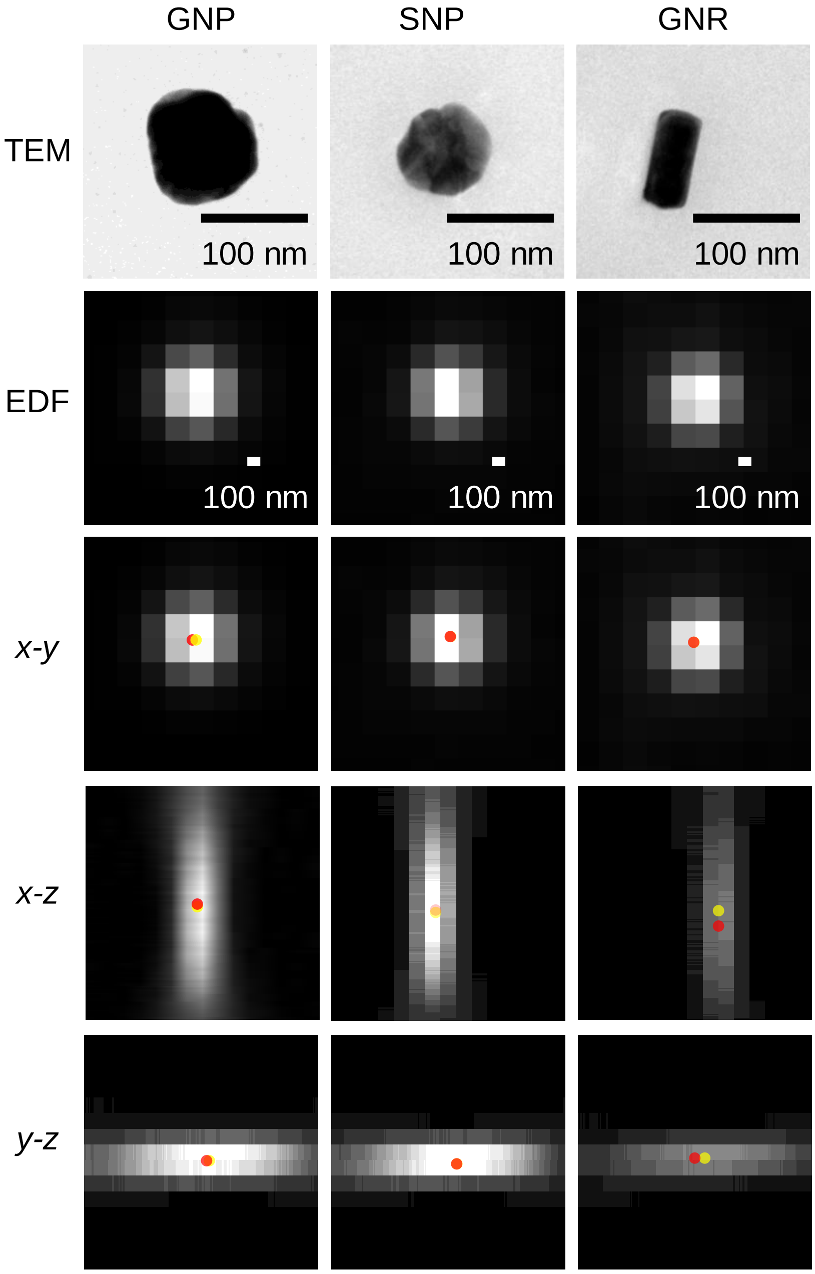

Supplementary Figure 6. The TEM images and EDF images (*xy*-, *xz*-, and *yz*-view) of GNP, SNP, and GNR. The red and yellow spots represent the centers calculated with the least-square and the least-cubic method, respectively.

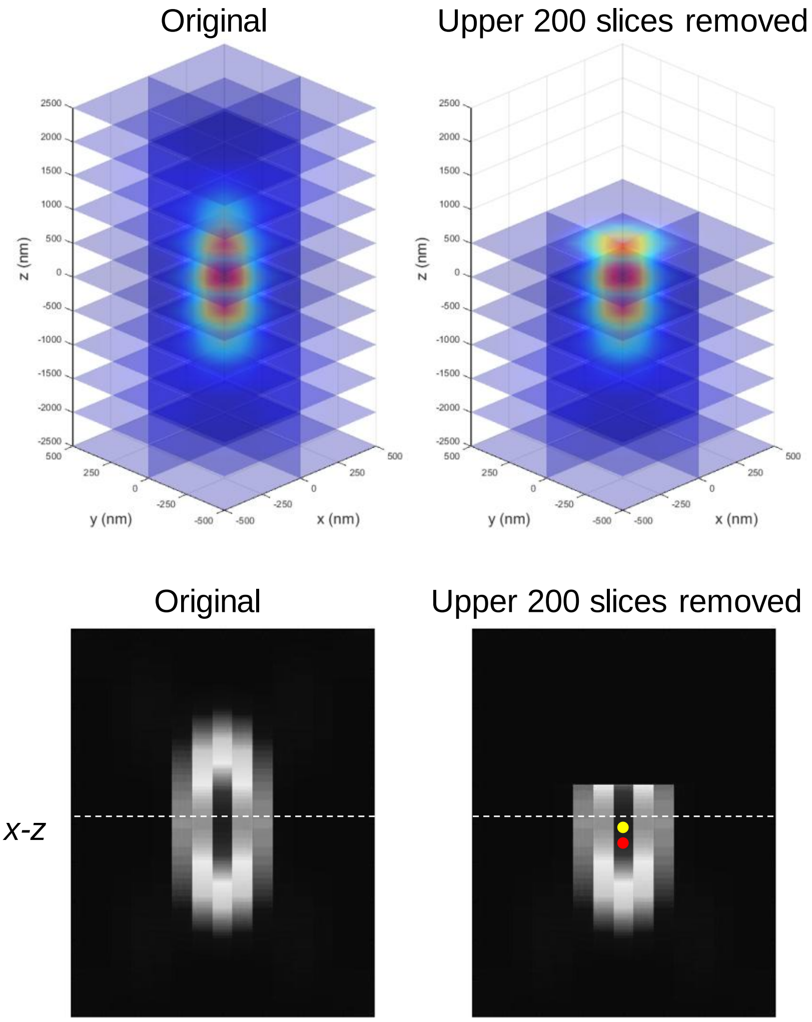

Supplementary Figure 7**.** The 3D and *xz*-view of simulated EDF images of GNP. The white line represents the real center line in the *z*-direction (z = 0), the red and yellow spots represent the centers calculated using the least-square and least-cubic method, respectively.

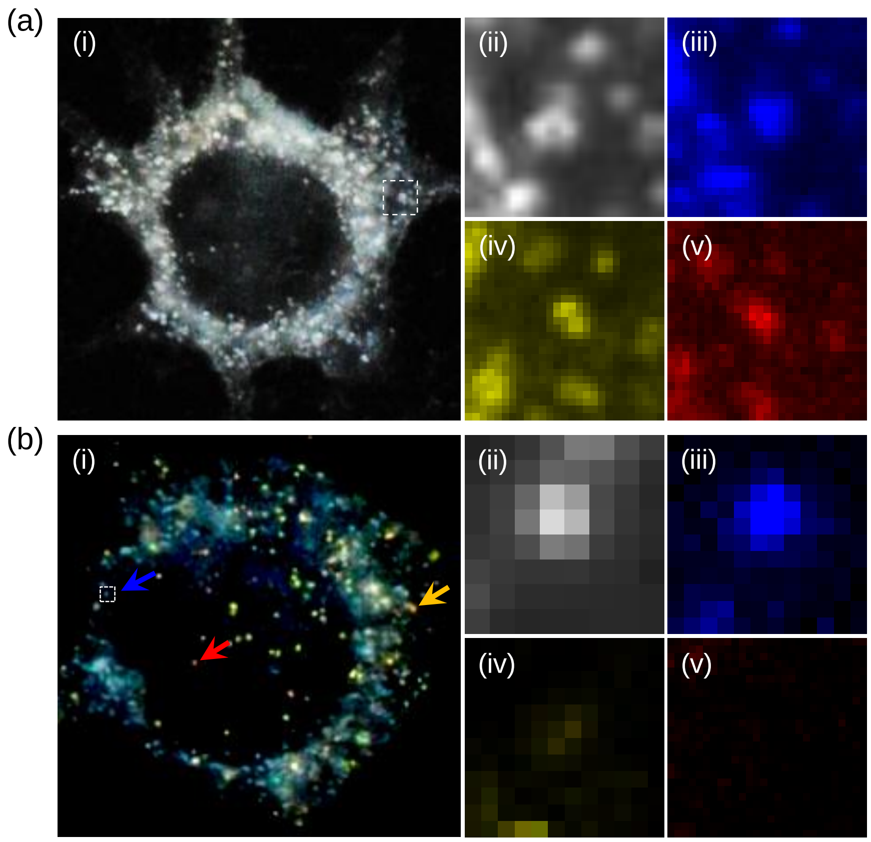

Supplementary Figure 8. EDF images of living single HEK293 cell with wavelength-modulation (a) before and (b) after labeling with NPs: (i) showed colored digital camera images; (ii), (iii), (iv) and (v) are the magnifications of the white-dotted rectangle area in (i) with EMCCD images and various bandpass filters. The yellow, blue and red arrows in (b) indicated the GNP, SNP and GNR, respectively. Except for nanoparticles, the cells in (a) and (b) were treated with identical procedures.

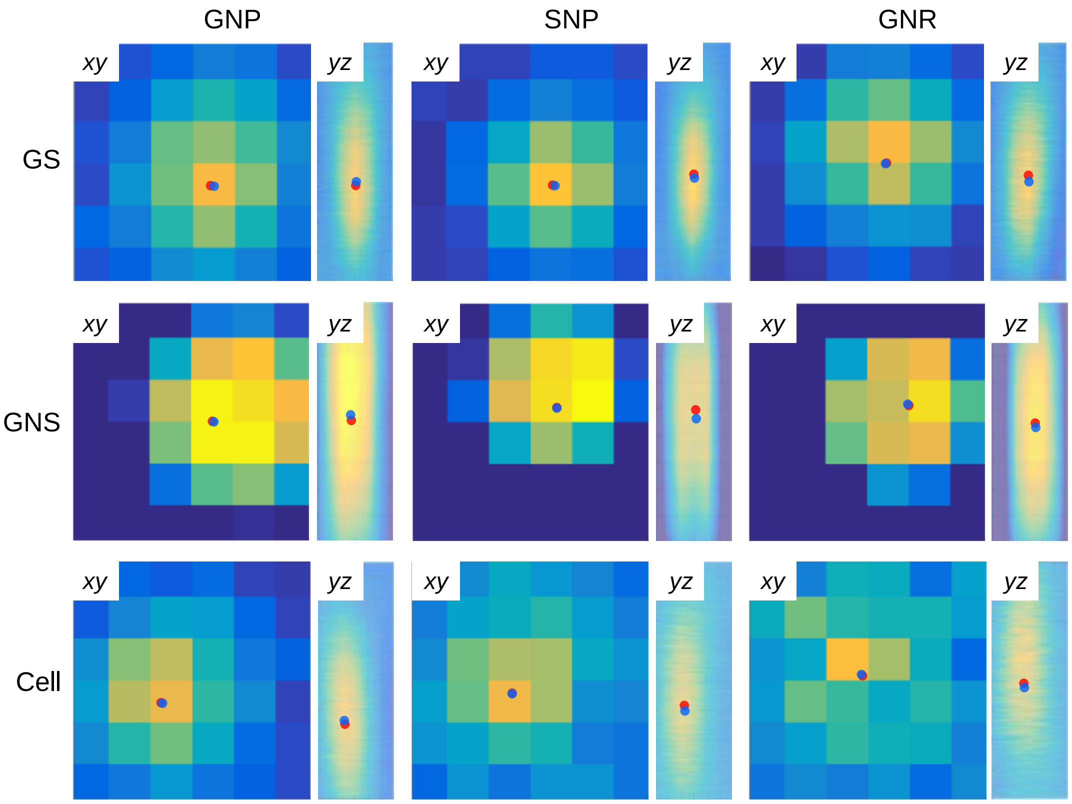

Supplementary Figure 9. Center positions of GNP, SNP and GNR on glass slide (GS), on gold nanospot (GNS) and in living HEK293 cell calculated by least-square (blue) and the least-cubic (red) method with *xy*- and *yz*-view.

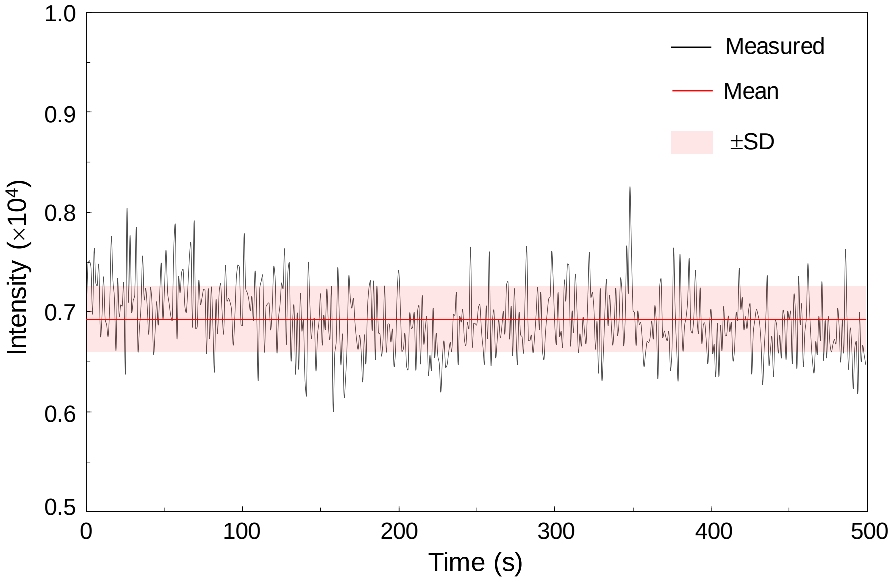

Supplementary Figure 10. The scattering intensity-time profiles of GNP under EDF. Black line, measured intensity; red line, mean intensity value during the detection period; red area, standard deviation (SD) during the detection period.

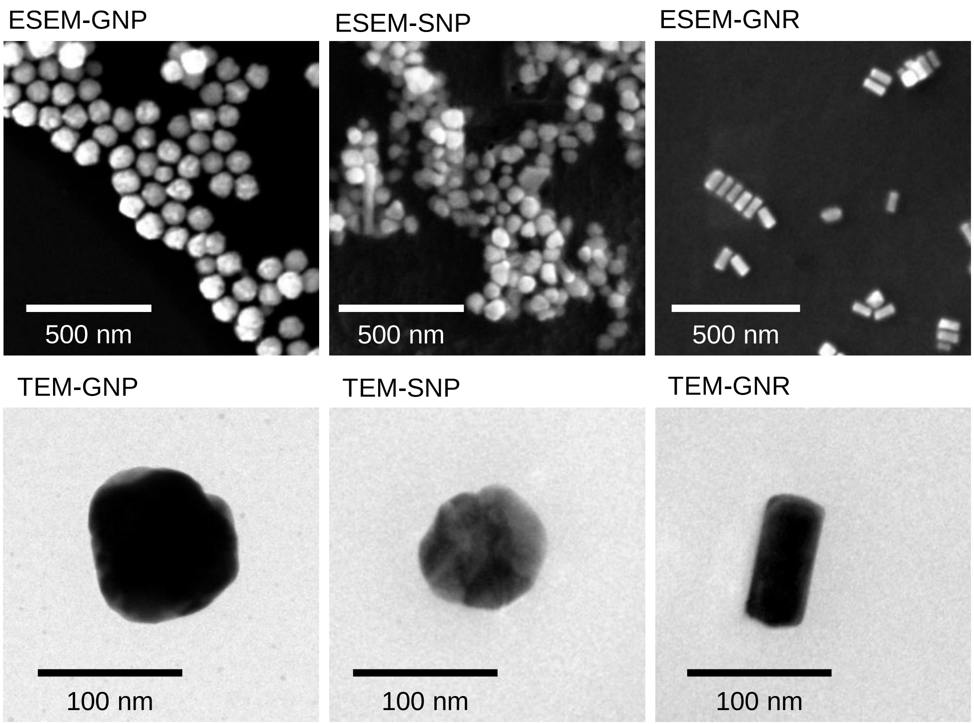

Supplementary Figure 11. ESEM and TEM images of 103-nm GNP, 80-nm SNP, and 40-nm GNR.

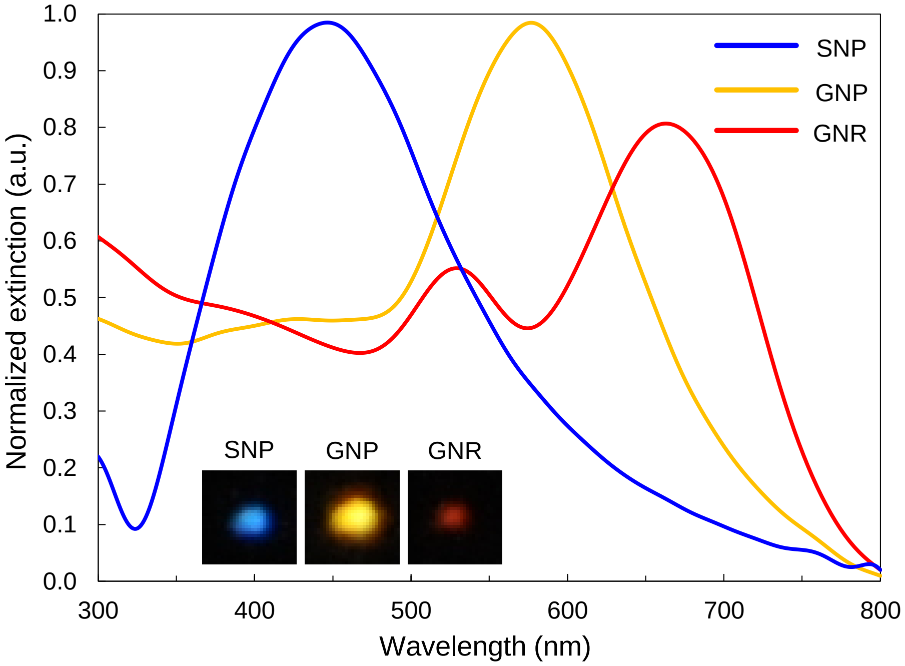

Supplementary Figure 12. UV-Vis spectra of GNP, SNP, and GNR and their EDF images in the single-particle state (inset).

(a)

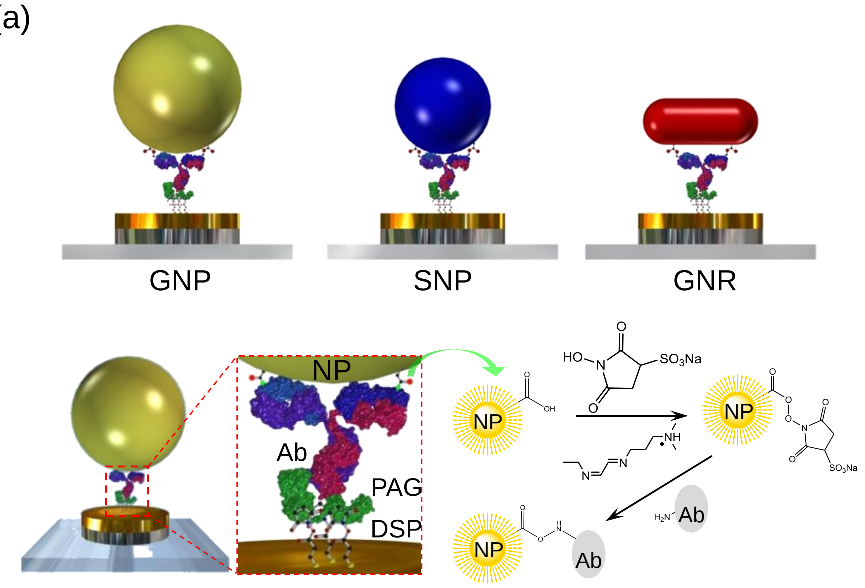

(b)

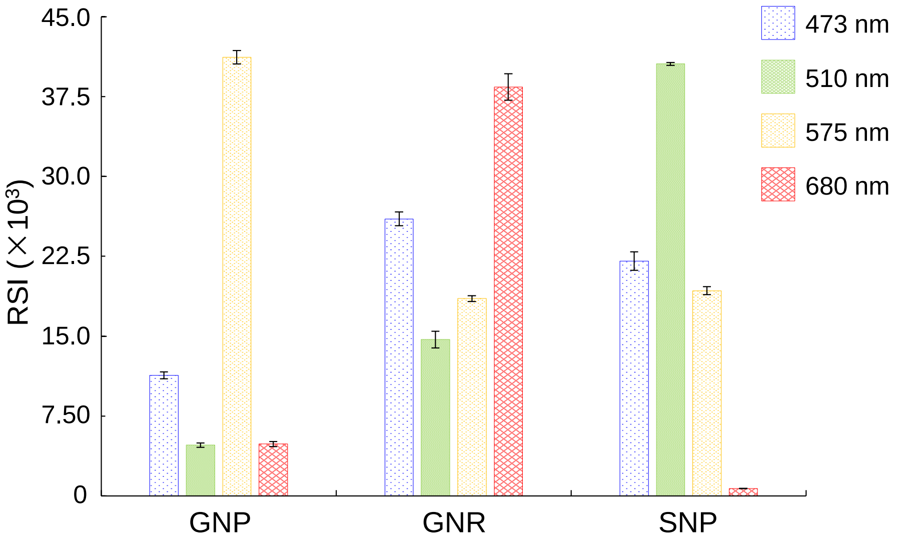

Supplementary Figure 13. (a) Conjugation of GNP, SNP, and GNR onto the gold nanospot via carbodiimide reaction. (b) Wavelength selections of NPs on the gold nanospot. NP: nanoparticle; Ab: antibody; PAG: protein A/G; DSP: dithiobis(succinimidyl propionate).

References

1. Wolf, E. Electromagnetic diffraction in optical Systems. I. an integral representation of the image field. *Proc. R. Soc. London A* **253**, 349-357 (1959).

2. Aspelmeier, T., Egner, A. & Munk, A. Modern Statistical Challenges in High-Resolution Fluorescence Microscopy. *Annu. Rev. Stat. Appl.* **2**, 163-202 (2015).

3. Kirshner, H., Aguet, F., Sage, D. & Unser, M. 3-D PSF fitting for fluorescence microscopy: implementation and localization application. *J. Microsc.* **249**, 13-25 (2013).
